# Profile of patients with genitourinary anomalies treated in a clinical genetics service in the Brazilian unified health system

**DOI:** 10.1016/j.rppede.2015.06.024

**Published:** 2016

**Authors:** Ilanna Fragoso Peixoto Gazzaneo, Camila Maia Costa de Queiroz, Larissa Clara Vieira Goes, Victor José Correia Lessa, Reinaldo Luna de Omena, Diogo Lucas Lima do Nascimento, Reginaldo José Petroli, Susane Vasconcelos Zanotti, Isabella Lopes Monlleó

**Affiliations:** a Universidade Federal de Alagoas (Ufal), Maceió, AL, Brazil; b Universidade Estadual de Ciências da Saúde de Alagoas (Uncisal), Maceió, AL, Brazil

**Keywords:** Genitalia, Sexual differentiation/genetics, Etiology

## Abstract

**Objective:**

To describe the profile of patients with genitourinary abnormalities treated at a tertiary hospital genetics service.

**Methods:**

Cross-sectional study of 1068 medical records of patients treated between April/2008 and August/2014. A total of 115 cases suggestive of genitourinary anomalies were selected, regardless of age. A standardized clinical protocol was used, as well as karyotype, hormone levels and genitourinary ultrasound for basic evaluation. Laparoscopy, gonadal biopsy and molecular studies were performed in specific cases. Patients with genitourinary malformations were classified as genitourinary anomalies (GUA), whereas the others, as Disorders of Sex Differentiation (DSD). Chi-square, Fisher and Kruskal–Wallis tests were used for statistical analysis and comparison between groups.

**Results:**

80 subjects met the inclusion criteria, 91% with DSD and 9% with isolated/syndromic GUA. The age was younger in the GUA group (*p*<0.02), but these groups did not differ regarding external and internal genitalia, as well as karyotype. Karyotype 46,XY was verified in 55% and chromosomal aberrations in 17.5% of cases. Ambiguous genitalia occurred in 45%, predominantly in 46,XX patients (*p*<0.006). Disorders of Gonadal Differentiation accounted for 25% and congenital adrenal hyperplasia, for 17.5% of the sample. Consanguinity occurred in 16%, recurrence in 12%, lack of birth certificate in 20% and interrupted follow-up in 31% of cases.

**Conclusions:**

Patients with DSD predominated. Ambiguous genitalia and abnormal sexual differentiation were more frequent among infants and prepubertal individuals. Congenital adrenal hyperplasia was the most prevalent nosology. Younger patients were more common in the GUA group. Abandonment and lower frequency of birth certificate occurred in patients with ambiguous or malformed genitalia. These characteristics corroborate the literature and show the biopsychosocial impact of genitourinary anomalies.

## Introduction

Genitourinary abnormalities (GUA) represent 35–45% of birth defects and include a wide range of structural abnormalities of the urinary and reproductive tracts, whose collective occurrence reflects their embryological origin and common genetic control.^1^^–^ 3 The clinical spectrum extends from minor anomalies such as glandular hypospadias to severe conditions such as bladder exstrophy. The clinical presentation may be isolated or associated with other anatomical defects and present syndromic conditions. The etiology comprises genetic causes resulting from chromosomal, monogenic or multifactorial abnormalities, not genetic, associated with exposure to teratogens, and there also the unknown causes.^1^^,^ 4

Disorders of Sex Differentiation (DSD) are a special group of GUA in which the development of genetic, gonadal or anatomical sex is atypical or incongruous. Clinical manifestations range from classical genital ambiguity, manifested at birth, to infertility in adults.^4^^,^ 6 ^–^ 11 Clinical heterogeneity and the use of different inclusion criteria, collecting methods, coding and recording of GUA account for wide variations in prevalence. With the exception of minor abnormalities, such as isolated hypospadias with a prevalence of 1:250 live births, some disorders may be as rare as 1:100,000, as in cloacal exstrophy.^12^^,^ 13 In the SDD group, a global prevalence of 1–2:10,000 births is assumed,^2^^,^ 4 ^,^ 9 ^,^ 11 which put these conditions in the group of so-called rare diseases, recent focus of health care policies in genetics in the National Health System (Sistema Único de Saúde – SUS).^14^

The management of patients with GUA requires a multidisciplinary approach in view of the underlying complex surgical, endocrine, genetic, social, psychological, and ethical issues.^4^^,^ 6 ^–^ 10 All these aspects make GUA an important epidemiological and clinical challenge. So, with the prospect of subsidizing health care improvement proposals in this area, the objective of this study was to describe the clinical profile of patients with GUA treated in clinical genetics service at a tertiary hospital in the SUS.

## Method

Descriptive and cross-sectional study based on analysis of 1068 digital medical records of patients seen in the Serviço de Genética Clínica of the Hospital Universitário Professor Alberto Antunes of the Universidade Federal de Alagoas (SGC/HUPAA/UFAL) between April 2008 and August 2014. This service, unique in the state, serves the entire SUS demand. The cases are referred via Coordenação de Regulação da Assistência, with an average volume of eight new cases per week. Patients under the age of 18 who have morphogenesis defects with or without association with psychomotor retardation/intellectual disabilities comprise the predominant group.

A total of 115 eligible cases were mapped from the search of the following descriptors, regardless of age at first medical consultation: genital ambiguity; micropenis; hypospadias (any grade); posterior labial fusion; gynecomastia; cryptorchidism; clitoromegaly; primary amenorrhea; secondary sexual underdevelopment; gonadal dysgenesis. Cases with incomplete description of the external genital morphology, karyotype absence, and conclusive diagnosis of conditions outside the GUA spectrum, such as inguinal hernias and Noonan syndrome, were excluded. The final sample consisted of 80 subjects.

Data were obtained using the own protocol of the GUA clinic consisting of history of present complaint and gestational history, genetic and environmental risk factors, and general physical examination with emphasis on genital manifestations. This protocol was established in 2009 and applied prospectively to 70 (87%) cases. Data from the remaining 10 (13%) patients treated in 2008 were obtained from the digital medical record of the service.

Micropenis was defined as penile length less than 2.5 standard deviations for age; cryptorchidism, as extra-scrotal position of the testis; and genital ambiguity were classified according to Prader's criteria^5^: P1 – Genitalia indistinguishable from female, except for enlarged phallus; P2 – Phallus further enlarged, labioscrotal fusion and without urogenital sinus; P3 – Phallus of penile appearance, almost total labioscrotal fusion and perineal opening of the urogenital sinus; P4 – Enlarged phallus, complete labioscrotal fusion and penoscrotal urogenital sinus opening; P5 – Phallus of penile appearance, complete labioscrotal fusion, urogenital sinus opening in the body of the phallus or balanic area.^15^

The basic complementary evaluation included peripheral blood karyotype with GTG banding and band resolutions of 400–450. At least 40 metaphases per patient were analyzed in the Human Cytogenetics Laboratory at the Universidade Estadual de Ciências da Saúde de Alagoas (UNCISAL), in addition to directed hormonal tests, ultrasound of the genitourinary tract (GUT) and, if necessary, laparoscopy and gonadal biopsy.

Investigation of the following genes was done in partnership with the Grupo Interdisciplinar de Estudos da Determinação e Diferenciação do Sexo (GIEDDS) and Centro de Biologia Molecular e Engenharia Genética (CBMEG) of the Universidade Estadual de Campinas (UNICAMP): *androgen receptor* (AR), *nuclear receptor subfamily* 5, *group* A, *member* 1 (NR5A1); *steroid*-5*-alpha-reductase, alpha polypeptide* 2 (SRD5A2); *sex determining region* Y (SRY); *cytochrome* P450, *family* 21, *subfamily* A, *polypeptide* 2 (CYP21A2), and *hydroxysteroid* (17-beta) *dehydrogenase* 3 (HSD17B3).

Cases of genitourinary malformations were classified as genitourinary defects (GUD) and the others as Disorders of Sex Differentiation (DSD). The independent variables and their categories of analysis include the demographic and clinical characteristics and the genetic characteristics. The collected demographic and clinical characteristics were: external genital morphology (ambiguous, feminine appearance, male appearance, or malformed), GUT findings (Müllerian derivatives, Wolffian derivatives, renal anomalies), age (<1; 1–9; 10–19; >19 years), how the child is raised (family definition of social gender role as male, female, or not defined, regardless of civil registration), civil registration (male, female, unregistered), follow-up interruption/interruption reasons (change of address, death, withdrawal). Genetic characteristics evaluated were: inbreeding, recurrence and maternal age ≥35 years (yes or no), karyotype (46,XY; 46,XX; other), presence of pathogenic mutation/polymorphism in the studied genes (yes or no).

Data were tabulated and analyzed using Microsoft Excel and Epi-Info™ version 3.5.2 softwares. Descriptive analysis with frequency distribution, measures of central tendency, and dispersion was performed. Fisher's exact test and chi-square were used for analysis of categorical variables and Kruskal–Wallis test for equality of means. A significance level of 5% (*p*<0.05) was considered. This study had ethical approval on 09/10/2013 (CAAE 17197113.8.0000.5013; Opinion 390.134).

## Results

Among 80 GUA subjects in the sample, 73 (91%) were defined as having DSD diagnosed and the other as having GUD alone or syndromic. Table 1 shows the distribution of general characteristics of subjects according to these groups.

**Table 1:** Distribution of demographic, clinical, and cytogenetic characteristics of subjects in relation to disorder group.

Characteristics		Genitourinary defects	Disorders of sexual differentiation	*p* -value
*Age in years, n (%)*				0.02 a
	0	6 (85.5)	32 (43.8)	
	1–9	1 (14.5)	16 (22)	
	10–19	–	18 (24.6)	
	>19	–	7 (9.6)	
	Mean±SD	0.14 (±0.37)	6.8 (±9.58)	
*External genital morphology, n (%)*				0.50 b
	Ambiguous	–	36 (49.3)	
	Male	3 (43)	20 (27.3)	
	Female	–	17 (23.4)	
	Malformed	4 (57)	–	
*Findings of the internal genitourinary tract, n (%)*				0.51 b
	Müller's derivatives	2 (28.5)	35 (48)	
	Wolf's derivatives	3 (43)	35 (48)	
	Renal anomalies	2 (28.5)	4 (5.4)	
*Karyotype , n* (%)				0.64 b
	46,XY	4 (57)	40 (54.7)	
	46,XX	2 (28.5)	20 (27.3)	
	Other	1 (14.5)	13 (18)	
*Absence of civil registration, n (%)*		3 (43)	13 (17.8)	0.13 b
*Reasons for withdrawal, n (%)*				0.56
	Death	2 (50)	3 (66.7)	
	Change of address	–	4 (19)	
	Withdrawal	2 (50)	14 (14.3)	

a Kruskal–Wallis.

b Fisher's exact test.

The age at presentation ranged from zero to 38, with 55 (69%) under six years. The average age was four months in GUD group and 6.8 years in DSD group (*p*<0.02). In the latter, there was a mean age distribution gradient in relation to the genital morphology, of 2.9±6.4 years in the ambiguous genitalia group, 6.5±10.2 years in the genitalia of male appearance group, and 5.5±9.1 years in the genitalia of female appearance group (*p*<0.01).

Ambiguous genitalia occurred in 36 (45%) cases, followed by genitalia of male appearance, female appearance, and malformed, without preferential distribution between DSD and GUD groups. GUT was evaluated in 75 patients. Wolffian derivatives were present in 38 (51%) subjects, six of whom had associated renal anomalies.

A 46,XY karyotype was found in 44 (55%) patients; 46,XX in 22 (27.5%); and chromosomal abnormalities in 14 (17.5%). There were no statistically significant differences between DSD and GUD groups for the presence of cytogenetic abnormalities.

Table 2 shows the distribution of external and internal genital manifestations in relation to karyotype. Genital ambiguity was more frequent in the 46,XX group (*p*<0.006). Six patients with Müllerian derivatives had Y chromosome demonstrable by cytogenetics or molecular technique.

**Table 2:** Distribution of subjects according to external and internal genitals characteristics in relation to karyotype.

Characteristics		46,XY n (%)	46,XX n (%)	Other n (%)	*p* -value Chi-square
*External genital morphology*					
*Ambiguous*		19 (43.2)	15 (68.1)	2 (14.2)	
	Prader 1	1 (2.3)	1 (4.5)	–	
	Prader 2	1 (2.3)	2 (9.1)	1 (7.1)	
	Prader 3	5 (11.3)	11 (50)	1 (7.1)	
	Prader 4	12 (27.3)	–	–	
	Prader 5	–	1 (4.5)	–	
*Male (micropenis, hypospadias and/or cryptorchidism)*		22 (50)	–	1 (7.1)	
*Female (normal, child or hypoplastic)*		2 (4.5)	5 (22.8)	10 (71.6)	
*Malformed*		1 (2.3)	2 (9.1)	1 (7.1)	
	Total	44 (55)	22 (27.5)	14 (17.5)	0.006
*Findings of internal genitourinary tract*					<0.001
	Müller's derivatives	3 (7.5)	22 (100)	12 (92.3)	
	Wolf's derivatives	37 (92.5)	–	1 (7.7)	

Table 3 shows the clinical categories. All found chromosomal abnormalities had pathogenic relationship with their phenotypes. Regarding the investigated genes, mutations/polymorphisms were found in 15 (18.8%) cases to date.

**Table 3:** Distribution of clinical categories according to external genital morphology, how the child is raised, and diagnostic methods used.

Clinical categories (n)	External genitalia	How the child is raised	Diagnostic methods		B. gonadal (n)
			Classical cytogenetics	Gene seq.	
Turner syndrome (9)	FA	F	45,X, 45,X/46,X,r(X)	–	–
DGP XX e XY (4)	FA	F	46,XX, 46,XY	NR5A1, SRY	–
Other gonadal dysgenesis (6)	P2–P4, MA	F, M, ND	46,XY, 45,X, a 45,X/46,XY, 45,X/47,XY+21	NR5A1, SRY	(3)
Ovarian-testicular SDD (1)	P3	F	46,XX	–	(1)
5α-reductase deficiency II (2)	FA, P4	F, M	46,XY	AR, SRD5A2	(1)
HSD17B3 deficiency (1)	P3	F	46,XY	AR, SRD5A2, HSD17B3	(1)
Under investigation (14)	MA, P2–P4	M, F	46,XY	AR, NR5A1, SRD5A2	(2)
Congenital adrenal hyperplasia (14)	P1–P5, MA	F, M	46,XX, 46,XY	CYP21A2	–
Hypogonadotropic hypogonadism (12)	MA, FA	M, F	46,XX, 46,XY	–	
Idiopathic or unenlightened (8)	P1–P4, MA	M, F	46,XY, 46,XX	AR, NR5A1, SRD5A2, HSD17B3	–
Genitourinary defects (7)	MA, MF	M, F	46,XX, 46,XY, 47,XX+18	–	
Other: 2	MA, FA	M, F	46,XY, 46,XY, der(22)t(X;22) (p11.4;p13)mat	SRY	(2)

a Y chromosome demonstrable by molecular technique.

P1, ambiguous genitalia Prader grade I; P2, ambiguous genitalia Prader grade II; P3, ambiguous genitalia Prader grade III; P4, ambiguous genitalia Prader grade IV; P5, ambiguous genitalia Prader grade V; MA, genitalia of male appearance±micropenis, glandular hypospadias, and cryptorchidism; FA, normal genitalia of female appearance, infantile or hypoplastic; MF: malformed genitalia; F, child raised as female; M, child raised as male; ND, not defined how the child is raised; AR, androgen receptor; NR5A1, nuclear receptor subfamily 5, group A, member 1; SRD5A2, steroid-5-alpha-reductase, alpha polypeptide 2; SRY, sex determining region Y; CYP21A2, cytochrome P450, family 21, subfamily A, polypeptide 2; HSD17B3, hydroxysteroid (17-beta) dehydrogenase 3.

Twenty-two (27.5%) patients remain under diagnostic investigation, only one of them with 46,XX karyotype. Among the rest, 14 have hormonal profile suggestive of defect in androgen synthesis or action and seven remain as idiopathic or unclear. No pathogenic mutation was found so far in the genes investigated in these subjects.

As a group, the disorders of gonadal differentiation were the most common, accounted for 20 (25%) cases. Among these, it was found the largest number of cytogenetic abnormalities and one of the lowest frequency of genital ambiguity.

Congenital adrenal hyperplasia was the most frequent nosological diagnosis in the sample (17.5%). In this, as in the group of defects in androgen synthesis or action, the highest frequency of genital ambiguity was found.

Consanguinity was found in 11 (16%) families, nine (12%) with recurrence of the disorder, all from the DSD group. Maternal age ≥35 years occurred in eight (10%) cases, only one of these in GUD group.

Sixteen (20%) children had no civil registration and only one had the social gender role not defined, without statistical differences between DSD and GUD groups (*p*<0.13) (Table 1). However, the lack of civil registration was higher in cases of ambiguity or external genital malformation (*p*<0.02).

There was interruption of outpatient follow-up in 25 (31%) cases, mainly by withdrawal. This result did not differ between GUD and DSD groups (Table 1), as well as between those living in the city or in the country (*p*<0.20). On the other hand, it was significantly lower in patients with genital ambiguity or malformed genitalia (*p*<0.01).

## Discussion

The SGC/HUPAA/UFAL is a referral service for people with birth defects in the state since 2003. A survey performed in 2007 of the actions involved in the care of patients with genital ambiguity in this hospital revealed some obstacles such as lack of information, difficult access to tests, discontinuation of follow-up, and disconnection between the services.^16^ The intention of changing this reality determined in 2008 the beginning of an integrated outpatient clinic of genetics and psychoanalysis in the SGC/HUPAA/UFAL.^17^ The results discussed here comes from the systematic care of patients provided at this clinic.

In this sample, there was predominance of DSD over isolated and syndromic GUD. Although subtle isolated GUD, such as glandular hypospadias, are very common in the population,^13^ this result is not surprising, as the common practice is not referring these patients for etiological investigation. On the other hand, the small number of cases with syndromic GUD may reflect the rarity and severe clinical course of these conditions usually associated with death. However, once recognized, these cases are usually referred for evaluation at an early age, as seen in this cohort.

DSD group was heterogeneous regarding the age of referral and clinical manifestations. The observed distribution gradient revealed that in infants and prepubescent there is a predominance of cases of genital ambiguity or signs suggestive of abnormal sexual differentiation (micropenis, bilateral cryptorchidism, microrquidia, hypospadias, clitoral hypertrophy, posterior labial fusion, palpable masses in the labioscrotal pouches or inguinal region). In adolescents and adults, the most prevalent manifestations were pubertal delay, atypical pubertal development, and infertility. These results corroborate the literature.^4^^,^ 6 ^–^ 8 ^,^ 11

Despite this, the wide age range in the group with ambiguous genitalia, which included teenagers and one adult, and the lack of prior treatment in six patients in this group drew attention. This result may reflect the underdiagnosis and underreporting of GUA, as well as the access difficulty and the disconnection between the health services in the state.^17^^,^ 18

The other clinical aspects evaluated—external and internal genital morphology and presence or absence of cytogenetic abnormalities—had similar behavior in GUD and DSD groups. Consistent with the literature, the analysis of these characteristics revealed that, although there was a predominance of subjects with ambiguous genitalia, derived from Wolff and 46,XY karyotype, none of these parameters alone may be considered for determination of diagnosis and treatment.^4^^,^ 6 ^–^ 8 ^,^ 10 ^,^ 11 ^,^ 19 ^–^ 21

The group with genital ambiguity is particularly illustrative of this situation. Note that in individuals with Prader 1–3 genitalia, all types of karyotype were detected, whereas in the Prader 4 group all had the XY sex pair and in the Prader 5 group the sex pair was XX. Furthermore, the Y chromosome was found in patients with normal female genitalia appearance and in patients with Müllerian derivatives, which supports the need for the use of various resources in the diagnostic approach of GUA.^4^^,^ 6 ^–^ 8 ^,^ 10 ^,11,^ 19 ^–^ 21

The establishment of the nosological diagnosis is admittedly a challenge, especially in the group with 46, XY karyotype, due to extensive clinical and laboratory overlap. Even with the advent of molecular biology techniques to approach several genes involved in sex differentiation, the frequency of cases with no diagnosis ranges from 33% to 80%.^4^^,^ 22 ^–^ 24 In the present study, the frequency of 46,XY cases without an established nosological diagnosis is in line with the literature data. The completion of the sequencing of genes selected to reassess this result is pending.

Groups of DGD and hypogonadotropic hypogonadism groups have in the karyotype and hormone profile significant resources for diagnosis definition. Among the DGD, the karyotype allows clarify Turner syndrome and mixed gonadal dysgenesis. Furthermore, gonadal biopsy, indicated according to strict criteria of cost-benefit, is instructive in cases of ovariotestis DSD and some gonadal dysgenesis. However, the presence of renal abnormalities and other birth defects in patients with hypogonadism should infer the possibility of Kallmann syndrome.^6^^,^ 11 All this characteristics were seen this study.

According to expected, among all diagnostic groups, the lowest frequency of disagreement between genital morphology, karyotype, and how the child is raised was seen in patients with hypogonadism and Turner syndrome, whereas the biggest disagreements occurred in the synthesis defects or androgen action and congenital adrenal hyperplasia.^4^^,^ 10 ^,^ 25 The latter, which corroborates the literature, was the most frequent nosological diagnosis.^26^^,^ 27

The high frequency of consanguinity and recurrence of the disorder in the DSD group complies with the recognized contribution of autosomal recessive conditions in the etiology of these disorders.^24^^,^ 26 ^,^ 28 It is noteworthy that the State of Alagoas has high frequency of consanguinity, which favors the appearance of rare disorders, as shown in some cases of this sample. Maternal age ≥35 years is an increased risk factor for chromosomal aneuploidies.^28^ This correlation can be inferred in two of the eight cases of this sample.

The biological complexity of sex differentiation in humans overlaps the challenging psychological, social, and ethical issues involved in these patients’ management. Thus, for therapeutic planning, one should take into account, in addition to etiologic diagnosis, how the child is raised, the anatomical conditions for external genital morphology repair, the possibility of spontaneous puberty, and fertility.^4^^,^ 6 ^–^ 8 ^,^ 10 ^,^ 11 The definition of the child's social gender role and civil registration are particularly important and should be analyzed comprehensively. In the present study, a family waited for clarification of diagnosis to define how the child would be raised and the civil registration and began follow-up with 65 days of life, without naming the child.

The family definition of how a child is raised regarding social gender reveals the attributes of a wish. A wish that involves the imaginary representations that are made of the child's anatomical sex and the place it occupies in the family.^29^ Thus, the way in which each subject will apprehend the body itself and the construction of psychosexuality follows the definition of how the child is raised, and not the anatomical and biological sex.^29^ In this regard, the study of one of the sample cases,^30^ based on the psychoanalytic theory, showed that given a proper name to a child puts the child, by virtue of that, in one of the sexes.

Regarding civil registration, it is important to note that it does not always follows the gender definition by the family, as one of the conducts in newborns is to guide parents to wait for the diagnostic conclusion to register the child. At the same time, the lack of civil registration makes the access to SUS difficult, as it makes it impossible to obtain the documents required for the national health card that provides access to tests and procedures. In this study, approximately 1/5 of the children came to the clinic without a civil registration; the majority was cases of genital ambiguity or malformed genitalia. This result suggests that the most significant factor in the decision to register the child is the external genital morphology and indicates the need to review the access criteria to SUS in cases in which the medical condition interferes in the civil registry.

The frequency of follow-up discontinuation was quite high in this study, particularly in cases of death and withdrawal. Deaths occurred in patients with congenital adrenal hyperplasia, related to adrenal crisis, and in babies born with multiple malformations of the GUD group, outcomes consistent with the literature considering the severity of conditions.^26^^–^ 28 The withdrawal cases include patients who did not return and change of telephone numbers and addresses. An interesting point to note is that the distance between the family home and the follow-up service did not affect the withdrawal. On the other hand, similar to what occurs with the civil registration, the external genital morphology was a determining factor. The lower withdrawal rates were observed in patients with genital ambiguity or malformation. These results highlight the psychosocial impact of external genital anatomy and indicate the need for greater investment in welcoming to families and efforts to maintain the follow-up of patients with less severe abnormalities in view of the implications of no treatment in puberty and adulthood.

The overall results allowed us to know the profile of GUA patients treated at the SGC/HUPAA/UFAL. The gathered information provide subsidies to improve the clinical protocols in order to facilitate and streamline the management of cases and approaches decisions based on evidence and specific health needs of individuals. Two important limitations of the study are the large number of cases whose follow-up was discontinued and cases still undiagnosed that, in this group, depends heavily on the investigation of genes not yet available in the SUS. Despite this, an aspect of great importance was the establishment of partnerships with research institutions to access to diagnostic tests. Locally, the challenge before us is the effective incorporation of other specialties in this proposal, in the perspective of a multidisciplinary approach in tune with the guidelines and principles of the Política Nacional de Atenção Integral às Pessoas com Doenças Raras.
