# Supplementary material for: Profile of patients with genitourinary anomalies treated in a clinical genetics service in the Brazilian unified health system
Source: Rev Paul Pediatr. 2016 Jan-Mar;34(1):91–8. doi: 10.1016/j.rppede.2015.06.024 (PMC4795727; doi:10.1016/j.rppede.2015.06.024)
Supplement: Translation

# Translation: Perfil de pacientes com anormalidades geniturinárias atendidos em serviço de genética clínica no sistema único de saúde

**Authors:** Ilanna Fragoso Peixoto Gazzaneo, Camila Maia Costa de Queiroz, Larissa Clara Vieira Goes, Victor José Correia Lessa, Reinaldo Luna de Omena, Diogo Lucas Lima do Nascimento, Reginaldo José Petroli, Susane Vasconcelos Zanotti, Isabella Lopes Monlleó

**Affiliations:** a Universidade Federal de Alagoas (Ufal), Maceió, AL, Brasil; b Universidade Estadual de Ciências da Saúde de Alagoas (Uncisal), Maceió, AL, Brasil

**Keywords:** Genitália, Diferenciação sexual/genética, Etiologia

## Abstract

**Objetivo:**

Descrever o perfil de pacientes com anormalidades geniturinárias atendidos em serviço de genética de hospital terciário.

**Métodos:**

Estudo transversal de 1.068 prontuários de pacientes atendidos entre abril/2008 e agosto/2014. Foram selecionados 115 casos sugestivos de anomalias geniturinárias, independentemente da idade. Usaram-se protocolo clínico padronizado, cariótipo, hormônios e ultrassonografia geniturinária para avaliação básica. Laparoscopia, biopsia gonadal e estudos moleculares foram feitos em casos específicos. Pacientes com malformações geniturinárias foram classificados como defeitos geniturinários (DGU), os demais, como distúrbios da diferenciação do sexo (DDS). Usaram-se qui-quadrado, Fisher e Kruskal-Wallis para análise estatística e comparação entre os grupos.

**Resultados:**

Preencheram os critérios de inclusão 80 sujeitos, 91% com DDS e 9% com DGU isolados/sindrômicos. A idade foi menor no grupo DGU (*p*<0,02), mas esses grupos não diferiram quanto a genitália externa, interna e cariótipo. Verificou-se cariótipo 46,XY em 55% e aberrações cromossômicas em 17,5% dos casos. Ambiguidade genital ocorreu em 45%, predominou em pacientes 46,XX (*p*<0,006). Distúrbios da diferenciação gonadal representaram 25% e hiperplasia adrenal congênita; 17,5% da amostra. Consanguinidade ocorreu em 16%, recorrência em 12%, ausência de registro civil em 20% e interrupção do seguimento em 31% dos casos.

**Conclusões:**

Predominaram pacientes com DDS. Ambiguidade genital e diferenciação sexual anômala foram mais frequentes entre recém-nascidos e pré-púberes. Hiperplasia adrenal congênita foi a nosologia mais prevalente. Pacientes mais jovens pertenciam ao grupo DGU. Menor frequência de registro civil e abandono ocorreram em pacientes com genitália ambígua ou malformada. Essas características corroboram a literatura e evidenciam o impacto biopsicossocial das anormalidades geniturinárias.

## Introdução

Anormalidades geniturinárias (AGU) representam 35 a 45% dos defeitos congênitos e compreendem um conjunto amplo de anomalias estruturais dos tratos urinário e reprodutivo, cuja ocorrência conjunta reflete sua origem embriológica e controle genético comum.^1^^-^ 3 O espectro clínico se estende desde anomalias *minor*, como hipospadia glandar, até quadros graves como extrofia de bexiga. A apresentação clínica pode ser isolada ou associada a outros defeitos anatômicos e compor quadros sindrômicos. A etiologia compreende causas genéticas decorrentes de anormalidades cromossômicas, monogênicas ou multifatoriais, e não genéticas, relacionadas à exposição a teratógenos. Há ainda as causas desconhecidas.^1^^,^ 4

Os distúrbios da diferenciação do sexo (DDS) constituem um grupo especial de AGU em que o desenvolvimento do sexo genético, gonadal ou anatômico é atípico ou incongruente. As manifestações clínicas abrangem desde ambiguidade genital clássica, manifestada ao nascimento, até infertilidade em adultos.^4^^,^ 6 ^-^ 11 A heterogeneidade clínica e o uso de diferentes critérios de inclusão, métodos de coleta, codificação e registro das AGU são responsáveis por variações amplas de prevalência. Excluindo-se as anormalidades *minor*, como as hipospadias isoladas com prevalência de 1:250 nascidos vivos, alguns distúrbios podem ser tão raros quanto 1:100.000, como ocorre na extrofia de cloaca.^12^^,^ 13 No grupo DDS, admite-se prevalência global de 1-2:10.000 nascimentos,^2^^,^ 4 ^,^ 9 ^,^ 11 o que posiciona essas condições no grupo das chamadas doenças raras, foco recente de políticas de atenção à saúde em genética no Sistema Único de Saúde (SUS).^14^

A atenção aos pacientes com AGU requer abordagem multidisciplinar em face das complexas questões cirúrgicas, endocrinológicas, genéticas, sociais, psicológicas e éticas subjacentes.^4^^,^ 6 ^-^ 10 Todos esses aspectos fazem das AGU um importante desafio epidemiológico e clínico. Assim, na perspectiva de subsidiar propostas de melhoria da atenção à saúde nessa área, o objetivo deste estudo é descrever o perfil clínico dos pacientes com AGU atendidos em serviço de genética clínica em hospital terciário no SUS.

## Método

Estudo descritivo e transversal baseado na análise de 1.068 prontuários digitais de pacientes atendidos no Serviço de Genética Clínica do Hospital Universitário Professor Alberto Antunes da Universidade Federal de Alagoas (SGC/HUPAA/UFAL) entre abril de 2008 e agosto de 2014. Esse Serviço, único no estado, atende a toda demanda do SUS. Os casos são referenciados via Coordenação de Regulação da Assistência, com volume médio de oito casos novos por semana. Pacientes até 18 anos que apresentam defeitos da morfogênese com ou sem associação com atraso neuropsicomotor/deficiência intelectual constituem o grupo predominante.

Foram mapeados 115 casos elegíveis a partir da busca dos seguintes descritores, independentemente da idade na primeira consulta: ambiguidade genital; micropênis; hipospádia (qualquer grau); fusão labial posterior; ginecomastia; criptorquidia; clitoromegalia; amenorreia primária; hipodesenvolvimento sexual secundário; disgenesias gonadais. Casos com descrição incompleta da morfologia genital externa, ausência de cariótipo e diagnóstico conclusivo de condições fora do espectro das AGU, como hérnias inguinais e síndrome de Noonan, foram excluídos. A amostra final foi de 80 sujeitos.

Os dados foram obtidos com o uso de protocolo próprio do ambulatório de AGU, que consiste de anamnese dirigida à queixa atual, antecedentes gestacionais, fatores de risco genéticos e ambientais e exame físico geral com ênfase nas manifestações genitais. Esse protocolo foi instituído em 2009 e aplicado prospectivamente a 70 (87%) casos. Os demais 10 (13%) pacientes, atendidos em 2008, tiveram seus dados obtidos a partir do prontuário digital do serviço.

Micropênis foi definido como tamanho peniano abaixo de 2,5 desvios-padrão para a idade; criptorquidia, como localização extraescrotal do testículo; e ambiguidade genital foi classificada de acordo com critérios de Prader:^5^ P1 – Genitália indistinguível da feminina, exceto pelo aumento do falo; P2 – Aumento maior do falo, fusão labioescrotal posterior e sem seio urogenital; P3 – falo de aspecto peniano, fusão labioescrotal quase total e abertura perineal do seio urogenital; P4 – Falo aumentado, fusão labioescrotal total e abertura do seio urogenital penoescrotal; P5 – Falo de aspecto peniano, fusão labioescrotal total, abertura do seio urogenital no corpo do falo ou balânica.^15^

A avaliação complementar básica compreendeu cariótipo de sangue periférico com bandeamento GTG e resolução de 400-450 bandas. Foram analisadas, no mínimo, 40 metáfases por paciente, feitas no Laboratório de Citogenética Humana da Universidade Estadual de Ciências da Saúde de Alagoas (Uncisal); exames hormonais dirigidos, ultrassonografia do trato geniturinário (TGU) e, quando necessário, laparoscopia e biópsia gonadal.

Estudo dos seguintes genes foi feito em parceria com o Grupo Interdisciplinar de Estudos da Determinação e Diferenciação do Sexo (GIEDDS) e Centro de Biologia Molecular e Engenharia Genética (CBMEG) da Universidade Estadual de Campinas (Unicamp): a*ndrogen receptor* (AR), *nuclear receptor subfamily* 5, *group* A, *member* 1 (NR5A1); *steroid-*5*-alpha-reductase, alpha polypeptide* 2 (SRD5A2); *sex determining region* Y (SRY); *cytochrome* P450, *family* 21, *subfamily* A, *polypeptide* 2 (CYP21A2) *e hydroxysteroid* (17-beta) *dehydrogenase* 3 (HSD17B3).

Casos de malformações geniturinárias foram classificados como defeitos geniturinários (DGU) e os demais, como distúrbios da diferenciação do sexo (DDS). As variáveis independentes e suas respectivas categorias de análise compreenderam as características demográficas e clínicas e as características genéticas. As características demográficas e clínicas coletadas foram: morfologia genital externa (ambígua, aparência feminina, aparência masculina ou malformada), achados do TGU (derivados de Müller, derivados de Wolff, anomalias renais), idade (<1; 1-9; 10-19; >19 anos), sexo de criação (atribuição familiar de sexo social masculino, feminino ou não definido, independentemente do registro civil), registro civil (masculino, feminino, não registrado), interrupção do seguimento/motivo da interrupção (mudança de domicílio, óbito, abandono do seguimento). Já as características genéticas avaliadas foram: consanguinidade, recorrência e idade materna ≥35 anos (sim ou não), cariótipo (46,XY; 46,XX; Outros), presença de mutação patogênica/polimorfismo nos genes estudados (sim ou não).

Os dados foram tabulados e analisados por meio dos programas Microsoft Excel e Epi-Info™ versão 3.5.2. Foi feita análise descritiva com distribuição de frequências, medidas de tendência central e dispersão. Teste exato de Fisher e qui-quadrado foram usados para análise de variáveis categóricas e Kruskal-Wallis para igualdade de médias. Adotou-se nível de significância de 5% (*p*<0,05). Esta pesquisa teve aprovação ética em 10/09/2013 (CAAE 17197113.8.0000.5013; parecer 390.134).

## Resultados

Entre 80 sujeitos com AGU que compuseram a amostra, 73 (91%) foram definidos com diagnóstico de DDS e os demais como DGU isolado ou sindrômico. A tabela 1 apresenta a distribuição das características gerais dos sujeitos de acordo com esses grupos.

**Tabela 1:** Distribuição das características demográficas, clínicas e citogenéticas dos sujeitos em relação ao grupo de distúrbio

Características		DGU	DDS	*p* ‐valor
*Idade em anos; n (%)*				0,02 a
	0	6 (85,5)	32 (43,8)	
	1–9	1 (14,5)	16 (22)	
	10–19	–	18 (24,6)	
	>19	–	7 (9,6)	
	Média±DP	0,14 (±0,37)	6,8 (±9,58)	
*Morfologia genital externa, n (%)*				0,50 b
	Ambígua	–	36 (49,3)	
	Masculina	3 (43)	20 (27,3)	
	Feminina	–	17 (23,4)	
	Malformada	4 (57)	–	
*Achados do TGU interno, n (%)*				0,51 b
	Derivados de Müller	2 (28,5)	35 (48)	
	Derivados de Wolf	3 (43)	35 (48)	
	Anomalias renais	2 (28,5)	4 (5,4)	
	Cariótipo, n (%)			0,64 b
	46,XY	4 (57)	40 (54,7)	
	46,XX	2 (28,5)	20 (27,3)	
*Outros*		1 (14,5)	13 (18)	
*Ausência de registro civil*		3 (43)	13 (17,8)	0,13 b
*Motivos de abandono*				0,56
	Óbito	2 (50)	3 (66,7)	
	Mudança de domicílio	–	4 (19)	
	Abandono do acompanhamento	2 (50)	14 (14,3)	

a Kruskal‐Wallis.

b Teste exato de Fisher.

DGU, defeito geniturinário; DDS, distúrbio da diferenciação do sexo; TGU, trato genitourinário; Abandono, abandono de seguimento.

A idade na consulta inicial variou de zero a 38 anos, com 55 (69%) abaixo de seis. A média de idade foi de quatro meses no grupo DGU e 6,8 anos no grupo DDS (*p*<0,02). Nesse último, observou-se gradiente de distribuição da média de idade em relação à morfologia genital, de 2,9±6,4 anos no grupo com ambiguidade genital, 6,5±10,2 anos no grupo com genitália de aparência masculina e 15,5±9,1 anos no grupo com genitália de aparência feminina (*p*<0,01).

A genitália ambígua ocorreu em 36 (45%) casos, seguida por genitália de aparência masculina, feminina e malformada, sem distribuição preferencial entre os grupos DDS e DGU. O TGU foi avaliado em 75 pacientes. Derivados de Wolff estavam presentes em 38 (51%) sujeitos, seis dos quais apresentavam anomalias renais associadas.

Cariótipo 46,XY foi demonstrado em 44 (55%) pacientes; 46,XX em 22 (27,5%); e anormalidades cromossômicas em 14 (17,5%). Não houve diferenças estatisticamente significativas entre os grupos DDS e DGU quanto à presença de anormalidades citogenéticas.

A tabela 2 apresenta a distribuição das manifestações genitais externas e internas em relação ao cariótipo. A ambiguidade genital foi mais frequente no grupo 46,XX (*p*<0,006). Seis pacientes com derivados de Müller tinham cromossomo Y demonstrável por técnica citogenética ou molecular.

**Tabela 2:** Distribuição dos sujeitos segundo características genitais externas e internas em relação ao cariótipo

Características		46,XY n (%)	46,XX n (%)	Outros n (%)	*p* ‐valor Qui‐quadrado
*Morfologia genital externa*					
*Ambígua*		19 (43,2)	15 (68,1)	2 (14,2)	
	Prader 1	1 (2,3)	1 (4,5)	–	
	Prader 2	1 (2,3)	2 (9,1)	1 (7,1)	
	Prader 3	5 (11,3)	11 (50)	1 (7,1)	
	Prader 4	12 (27,3)	–	–	
	Prader 5	–	1 (4,5)	–	
*Masculina (micropênis, hipospadia e/ou criptorquidia)*		22 (50)	–	1 (7,1)	
*Feminina (normal, infantil ou hipoplásica)*		2 (4,5)	5 (22,8)	10 (71,6)	
*Malformada*		1 (2,3)	2 (9,1)	1 (7,1)	
	Total	44 (55)	22 (27,5)	14 (17,5)	0,006
*Achados do trato geniturinário interno*					<0,001
	Derivados de Müller	3 (7,5)	22 (100)	12 (92,3)	
	Derivados de Wolf	37 (92,5)	–	1 (7,7)	

As categorias clínicas estão apresentadas na tabela 3. Todas as anormalidades cromossômicas encontradas tiveram correlação patogênica com os respectivos fenótipos. Quanto aos genes estudados, até o presente foram identificadas mutações/polimorfismos em 15 (18,8%) casos.

**Tabela 3:** Distribuição das categorias clínicas de acordo com morfologia genital externa, sexo de criação e métodos diagnósticos empregados

Categorias clínicas (n)	Genitália externa	Sexo de criação	Métodos diagnósticos		B. gonadal (n)
			Citogenética clássica	Seq. genes	
Síndrome de Turner (9)	AF	F	45,X, 45,X/46,X,r(X)	–	–
DGP XX e XY (4)	AF	F	46,XX, 46,XY	NR5A1, SRY	–
Outras disgenesias gonadais (6)	P2–P4, AM	F, M, ND	46,XY, 45,X, a 45,X/46,XY, 45,X/47,XY+21	NR5A1, SRY	(3)
DDS ovário‐testicular (1)	P3	F	46,XX	–	(1)
Deficiência de 5 α redutase II (2)	AF, P4	F, M	46,XY	AR, SRD5A2	(1)
Deficiência de HSD17B3 (1)	P3	F	46,XY	AR, SRD5A2, HSD17B3	(1)
Em investigação (14)	AM, P2–P4	M, F	46,XY	AR, NR5A1, SRD5A2	(2)
Hiperplasia adrenal congênita (14)	P1–P5, AM	F, M	46,XX, 46,XY	CYP21A2	–
Hipogonadismo hipogonadotrófico (12)	AM, AF	M, F	46,XX, 46,XY	–	
Idiopáticos ou não esclarecidos (8)	P1–P4, AM	M, F	46,XY, 46,XX	AR, NR5A1, SRD5A2, HSD17B3	–
Defeitos geniturinários (7)	AM, MF	M, F	46,XX, 46,XY, 47,XX+18,	–	
Outros: 2	AM, AF	M, F	46,XY, 46,XY, der(22)t(X;22) (p11.4;p13)mat	SRY	(2)

a Cromossomo Y demonstrável por técnica molecular.

P1, genitália ambígua grau I de Prader; P2, genitália ambígua grau II de Prader; P3, genitália ambígua grau III de Prader; P4, genitália ambígua grau IV de Prader; P5, genitália ambígua grau V de Prader; AM, genitália de aparência masculina ± micropênis, hipospadia glandar e criptorquidia; AF, genitália de aparência feminina normal, infantil ou hipoplásica; MF: genitália malformada; F, sexo de criação feminino; M, sexo de criação masculino; ND, sexo de criação não definido; AR, *androgen receptor* ; NR5A1, *nuclear receptor subfamily* 5, *group* A, *member* 1; SRD5A2, *steroid‐5‐alpha‐reductase, alpha polypeptide* 2; SRY, *sex determining region* Y; CYP21A2, *cytochrome* P450, *family* 21, *subfamily* A, *polypeptide* 2; HSD17B3, *hydroxysteroid* (17‐beta) *dehydrogenase* 3.

Vinte e dois (27,5%) pacientes permanecem sob investigação diagnóstica, apenas um desses com cariótipo 46,XX. Dentre os demais, 14 têm perfil hormonal sugestivo de defeito de síntese ou ação dos andrógenos e sete permanecem como idiopáticos ou não esclarecidos. Nenhuma mutação patogênica foi encontrada até o momento nos genes estudados nesses indivíduos.

Como grupo, os distúrbios da diferenciação gonadal foram os mais frequentes, corresponderam a 20 (25%) casos. Entre esses foi encontrado o maior número de anormalidades citogenéticas e uma das menores frequências de ambiguidade genital.

A hiperplasia adrenal congênita foi o diagnóstico nosológico mais prevalente na amostra (17,5%). Nesse, assim como no grupo dos defeitos de síntese e ação de andrógenos, foram encontradas as frequências mais elevadas de ambiguidade genital.

Onze (16%) famílias apresentavam consanguinidade e nove (12%), recorrência do distúrbio, todas pertencentes ao grupo DDS. Idade materna ≥ 35 anos ocorreu em oito (10%) casos, apenas um desses no grupo DGU.

Dezesseis (20%) crianças não tinham registro civil e apenas uma não tinha sexo de criação definido, sem diferenças estatísticas entre os grupos DDS e DGU (*p*<0,13) (tabela 1). Todavia, a ausência de registro foi maior nos casos com ambiguidade ou malformação genital externa (*p*<0,02).

Houve interrupção do seguimento ambulatorial em 25 (31%) casos, principalmente por abandono do acompanhamento. Esse resultado não diferiu entre os grupos DGU e DDS (tabela 1) e tampouco quanto ao domicílio na capital ou no interior (*p*<0,20). Por outro lado, essa foi significativamente menor no grupo de pacientes com ambiguidade genital ou genitália malformada (*p*<0,01).

## Discussão

O SGC/HUPAA/UFAL é referência para atendimento a pessoas com defeitos congênitos no estado desde 2003. Pesquisa feita em 2007 sobre as ações envolvidas na atenção a pacientes com ambiguidade genital nesse hospital evidenciou alguns entraves como desinformação, dificuldade de acesso a exames, descontinuidade no acompanhamento e desarticulação entre os serviços.^16^ A intenção de modificar essa realidade determinou, em 2008, o início do ambulatório integrado de genética e psicanálise no SGC/HUPAA/UFAL.^17^ Os resultados aqui discutidos provém do atendimento sistematizado de pacientes feito nesse ambulatório.

Nessa amostra, observou-se predomínio dos DDS sobre os DGU isolados e sindrômicos. Embora os DGU isolados sutis, como as hipospádias glandares, sejam muito frequentes na população,^13^ esse resultado não surpreende, uma vez que a prática comum é não encaminhar esses pacientes para investigação etiológica. Por outro lado, o pequeno número de casos com DGU sindrômico pode refletir a raridade e curso clínico grave dessas condições, geralmente associadas a óbito. Todavia, uma vez reconhecidos, esses casos costumam ser encaminhados para avaliação em idade precoce, conforme observado nesta coorte.

O grupo DDS mostrou-se heterogêneo quanto à idade de encaminhamento e às manifestações clínicas. O gradiente de distribuição observado revelou que, em recém-nascidos e pré-púberes, predominaram os casos de ambiguidade genital ou com sinais sugestivos de diferenciação sexual anômala (micropênis, criptorquidia bilateral, microrquidia, hipospádia, hipertrofia clitoriana, fusão labial posterior, massas palpáveis em saliência labioescrotal ou região inguinal). Em adolescentes e adultos, as manifestações mais prevalentes foram atraso puberal, desenvolvimento puberal atípico e infertilidade. Esses resultados corroboram a literatura.^4^^,^ 6 ^-^ 8 ^,^ 11

A despeito disso, chamou atenção a ampla variação de idade no grupo com ambiguidade genital, que incluiu adolescentes e um adulto, e a ausência de tratamento prévio em seis pacientes desse grupo. Esse resultado pode refletir o subdiagnóstico e o sub-registro de AGU, além de dificuldades de acesso e desarticulação dos serviços de saúde no estado.^17^^,^ 18

Os demais aspectos clínicos estudados – morfologia genital externa e interna e presença ou não de anormalidades citogenéticas – tiveram comportamento semelhante nos grupos DGU e DDS. Em concordância com a literatura, a análise dessas características revelou que, embora tenha ocorrido predomínio de sujeitos com genitália ambígua, derivados de Wolff e cariótipo 46,XY, nenhum desses parâmetros isoladamente pode ser considerado para a definição do diagnóstico e do tratamento.^4^^,^ 6 ^-^ 8 ^,^ 10 ^,^ 11 ^,^ 19 ^-^ 21

O grupo com ambiguidade genital é particularmente ilustrativo dessa situação. Note-se que em indivíduos com genitália Prader 1-3 foram detectados todos os tipos de cariótipo, enquanto no grupo Prader 4 todos tinham par sexual XY e no grupo Prader 5 o par sexual era XX. Além disso, cromossomo Y foi encontrado em pacientes com genitália de aparência feminina normal e em pacientes com derivados de Müller, o que corrobora a necessidade de uso de vários recursos na abordagem diagnóstica das AGU.^4^^,^ 6 ^-^ 8 ^,^ 10 ^,^ 11 ^,^ 19 ^-^ 21

O estabelecimento do diagnóstico nosológico é reconhecidamente um desafio, especialmente no grupo com cariótipo 46,XY, devido à extensa sobreposição clínica e laboratorial. Mesmo com o advento de técnicas de biologia molecular para abordagem de vários genes envolvidos na diferenciação do sexo, a frequência de casos sem diagnóstico varia de 33% a 80%.^4^^,^ 22 ^-^ 24 No presente estudo, a frequência de casos 46,XY sem diagnóstico nosológico estabelecido concorda com os dados da literatura. Aguarda-se a conclusão do sequenciamento dos genes selecionados para reavaliar esse resultado.

Os grupos de DDG e hipogonadismo hipogonadotrófico têm no cariótipo e no perfil hormonal importantes recursos para a definição do diagnóstico. Entre os DDG, o cariótipo permite esclarecer síndrome de Turner e disgenesias gonadais mistas. Além disso, a biópsia gonadal, indicada de acordo com critérios estritos de custo/benefício, é elucidativa em casos de DDS ovariotesticular e em algumas disgenesias gonadais. Já a presença de anomalias renais e outros defeitos congênitos em pacientes com hipogonadismo hipogonadotrófico deve remeter à possibilidade de síndrome de Kallmann.^6^^,^ 11 Todas essa características foram observadas neste estudo.

De acordo com o esperado, entre todos os grupos diagnósticos, a menor frequência de discordância entre morfologia genital, cariótipo e sexo de criação foi observada em pacientes com hipogonadismo hipogonadotrófico e síndrome de Turner, ao passo que as maiores discordâncias ocorreram nos defeitos da síntese ou ação dos andrógenos e na hiperplasia adrenal congênita.^4^^,^ 10 ^,^ 25 Essa última, que corrobora a literatura, figurou como o diagnóstico nosológico mais frequente.^26^^,^ 27

A elevada frequência de consanguinidade e recorrência do distúrbio no grupo DDS está de acordo com a reconhecida contribuição das condições autossômicas recessivas na etiologia desses distúrbios.^24^^,^ 26 ^,^ 28 Destaca-se que o Estado de Alagoas apresenta frequências elevadas de consanguinidade, o que favorece o aparecimento de distúrbios raros, conforme se observa em alguns casos dessa amostra. A idade materna ≥35 anos é um fator que eleva o risco de aneuploidias cromossômicas.^28^ Essa correlação pode ser inferida em dois entre oito casos da presente amostra.

A complexidade biológica da diferenciação do sexo na espécie humana se sobrepõe a desafiadoras questões psicológicas, sociais e éticas envolvidas no manejo desses pacientes. Assim, para o planejamento terapêutico devem-se levar em consideração, além do diagnóstico etiológico, o sexo de criação, as condições anatômicas para correção da morfologia genital externa, a possibilidade de puberdade espontânea e fertilidade.^4^^,^ 6 ^-^ 8 ^,^ 10 ^,^ 11 A definição do sexo de criação e o registro civil da criança são aspectos particularmente importantes e devem ser analisados de modo abrangente. No presente estudo, uma família aguardou o esclarecimento do diagnóstico para definir o sexo de criação e registro civil e iniciou o acompanhamento com 65 dias de vida, sem nomeação.

A definição do sexo de criação por uma família inscreve as insígnias de um desejo. Desejo que implicará as representações imaginárias que se fazem do sexo anatômico da criança e do lugar que ocupa na família.^29^ Assim, o modo como cada sujeito irá apreender o corpo próprio e a construção da psicossexualidade seguem a definição do sexo de criação, e não o sexo anátomo-biológico.^29^ A esse respeito, o estudo de um dos casos da amostra,^30^ fundamentado na teoria psicanalítica, evidenciou que a atribuição do nome próprio situa a criança, por força disso, em um dos sexos.

Com relação ao registro civil, é importante destacar que esse nem sempre acompanha a definição do sexo de criação, uma vez que uma das condutas em recém-nascidos é orientar os pais a esperar a conclusão diagnóstica para registrar a criança. Ao mesmo tempo, a ausência do registro civil dificulta o acesso ao SUS, uma vez que inviabiliza a obtenção de documentos exigidos para confecção do cartão nacional de saúde, o qual possibilita o acesso a exames e procedimentos. Neste estudo, aproximadamente 1/5 das crianças chegou ao atendimento sem registro civil, a maioria constituída de casos de ambiguidade genital ou genitália malformada. Esse resultado sugere que o fator mais significativo na decisão de registrar a criança é a morfologia genital externa e indica a necessidade de revisar os critérios de acesso ao SUS nos casos em que a condição clínica interfere no registro civil.

A frequência de interrupção de seguimento foi bastante elevada neste estudo, com destaque para os casos de óbito e abandono. Os óbitos ocorreram em pacientes com diagnóstico de hiperplasia adrenal congênita, relacionados a crises adrenais, e em recém-nascidos com múltiplas malformações do grupo DGU, desfechos concordantes com a literatura considerando a gravidade dos quadros.^26^^-^ 28 Os casos de abandono incluem pacientes que não retornaram e cujos telefones e endereços mudaram. Um aspecto interessante é observar que a distância entre o domicílio da família e o serviço no qual ocorreu o acompanhamento não interferiu no abandono do acompanhamento. Por outro lado, à semelhança do que ocorre com o registro civil, a morfologia genital externa foi fator determinante. As menores taxas de abandono foram observadas em pacientes com ambiguidade ou malformação genital. Esses resultados evidenciam o impacto psicossocial da anatomia genital externa e indicam a necessidade de maior investimento na acolhida às famílias e nos esforços para manter o acompanhamento de pacientes com anormalidades menos graves, em face das implicações da ausência de tratamento na puberdade e idade adulta.

O conjunto dos resultados permitiu conhecer o perfil dos pacientes com AGU atendidos no SGC/HUPAA/UFAL. As informações reunidas fornecem subsídios para aprimorar os protocolos clínicos na perspectiva de facilitar e agilizar a condução dos casos e a tomada de condutas baseadas em evidência e nas necessidades de saúde específicas dos sujeitos. Duas importantes limitações do estudo são o elevado número de casos cujo seguimento foi interrompido e de casos ainda sem diagnóstico, o que, nesse grupo, depende fortemente do estudo de genes ainda não disponível no SUS. A despeito disso, um aspecto de grande relevância foi o estabelecimento de parcerias com instituições de pesquisa para acesso a testes diagnósticos. Localmente, o desafio que se apresenta é a efetiva incorporação de outras especialidades nessa proposta, na perspectiva da abordagem multidisciplinar afinada com as diretrizes e os princípios da Política Nacional de Atenção Integral às Pessoas com Doenças Raras.
